# Immediate Physical Therapy following Total Joint Arthroplasty: Barriers and Impact on Short-Term Outcomes

**DOI:** 10.1155/2019/6051476

**Published:** 2019-04-08

**Authors:** Hunter Warwick, Andrew George, Claire Howell, Cynthia Green, Thorsten M. Seyler, William A. Jiranek

**Affiliations:** Department of Orthopaedic Surgery, Duke University Hospital, Durham, NC 27710, USA

## Abstract

**Background:**

Recent evidence suggests benefit to receiving physical therapy (PT) the same day as total joint arthroplasty (TJA), but relatively little is known about barriers to providing PT in this constrained time period. We address the following questions: (1) Are there demographic or perioperative variables associated with receiving delayed PT following TJA? (2) Does receiving immediate PT following TJA affect short-term outcomes such as length of stay, discharge disposition, or 30-day readmission?* Methods*. Primary TJA procedures at a single center were retrospectively reviewed. Immediate PT was defined as within eight hours of surgery. Demographic and perioperative variables were compared between patients who received immediate PT and those who did not. We identified an appropriately matched control group of patients who received immediate PT. Postoperative length of stay, discharge disposition, and 30-day readmissions were compared between matched groups.

**Results:**

In total, 2051 primary TJA procedures were reviewed. Of these, 226 (11.0%) received delayed PT. These patients had a higher rate of general anesthesia (25.2% versus 17.8%, p=0.006), later operative start time (13:26 [11:31-14:38] versus 9:36 [8:24-11:16], p<0.001), longer operative time (1.8 [1.5-2.2] versus 1.6 [1.4-1.8] hours, p=0.002), and higher overall caseload on the day of surgery (6 [4-9] versus 5 [4-8], p=0.002). A matched group of patients who received immediate PT was identified. There were no differences in postoperative length of stay or discharge disposition between matched immediate and delayed PT groups, but delayed PT (OR 4.54; 95% CI 1.61-12.84; p=0.004) was associated with a higher 30-day readmission rate.

**Conclusion:**

Barriers to receiving immediate PT following TJA included general anesthesia, later operative start time, longer operative time, and higher daily caseload. These factors present potential targets for improving the delivery of immediate postoperative PT. Early PT may help reduce 30-day readmissions, but additional research is necessary to further characterize this relationship.

## 1. Introduction

Total joint arthroplasty (TJA) is a highly successful surgical intervention, providing significant improvements in physical function and quality of life [1]. Given the rising demand and high costs associated with TJA [2, 3], an increasing focus is being placed on accelerated perioperative care to speed recovery, improve patient outcomes, and decrease costs [4, 5]. These programs emphasize preoperative patient education and counseling, multimodal perioperative analgesia, and aggressive postoperative rehabilitation [6].

Early postoperative physical therapy (PT) is a key component of accelerated perioperative care, and health systems are beginning to implement perioperative pathways that include early PT following TJA. However, there is little knowledge on the challenges associated with providing PT in this constrained time period. One study [7] identified late operative end time, defined as after 15:00, as a risk factor for not receiving PT on the same day as surgery. Still, 80% of patients who did not receive PT on the same day as surgery had an operative end time before 15:00, suggesting that there are additional factors at play. Identifying these barriers could hold implications for improving the delivery of early PT following TJA.

Several studies have demonstrated benefits of earlier postoperative PT after TJA, including decreased length of stay (LOS), reductions in cost, and clinical improvements in pain, range of motion, quadriceps and hamstring strength, gait, and balance [7–10]. However, several recent systematic reviews have questioned the quality of evidence supporting these benefits [11, 12]. Moreover, many of these studies examined enhanced perioperative pathways in general without investigating individual components such as early postoperative PT. Thus, it is important to examine the isolated effects of early PT on short-term postoperative outcomes.

In this study, we address the following questions: (1) Are there demographic or perioperative variables associated with receiving delayed PT following TJA? (2) Does receiving immediate PT following TJA affect short-term outcomes such as length of stay, discharge disposition, or 30-day readmission?

## 2. Methods

This retrospective study was performed at a single academic medical center from July 2015 to December 2017. All primary total hip and knee arthroplasty procedures performed at the center during this period were analyzed following appropriate Institutional Review Board approval. Patients below 18 years of age and patients undergoing nonelective TJA for traumatic or neoplastic etiologies were excluded from analysis.

The standard of care at our institution is for patients undergoing TJA to receive PT within eight hours of surgery. Thus, immediate PT was defined as being delivered within the eight-hour time period following the operation. Patients are encouraged to get out of bed and ambulate during the first visit, but each therapy session is tailored to an individual patient's needs and abilities and can range from bed exercises to walking stairs. Cases that did not receive immediate PT (delayed PT) were identified and compared to those that did (immediate PT).

Baseline and perioperative variables assessed included sex, age, body mass index (BMI), preoperative American Society of Anesthesiology (ASA) classification, insurance type, procedure performed, primary anesthesia type, procedure start time, operative time, and overall TJA caseload on the day of surgery. Information on specific comorbidities and postoperative complications was unavailable. Primary anesthesia type was classified as either general or regional. Regional anesthesia techniques included central neuraxial blockade, peripheral nerve blockade, and monitored anesthesia care. Patients receiving a combination of both general and regional anesthesia were classified as general. Operative time was from incision to closure. Caseload was defined as the total number of primary TJA cases performed at the institution on the day of the procedure. Location of and time to first PT session were also recorded for each procedure. Time to first PT session was defined as the time between leaving the operating room (OR) and the first PT session. For the Immediate PT group, we determined whether patients were able to ambulate during their initial PT session or not.

For the delayed PT group, medical records were reviewed to determine the reason for not receiving immediate PT. Several steps must occur in order for a patient to receive immediate PT. First, a physical therapist is assigned to each patient at the beginning of the day based on the operative schedule. Then, following surgery, anesthesia must sign off on the patient before 19:00, the order for PT must be activated by the nursing staff before 19:00, and the patient must agree to participate in PT. Our PT department does not target patients for immediate postoperative PT if anesthesia signs off or PT order activation occurs after 19:00. This sequence was assessed for each patient that did not receive immediate PT to determine the reason for delayed PT. If another reason was discovered on review, it was recorded. If all necessary steps were documented and no other reason was identified, it was classified as a “failure to see patient.” For these cases and those where PT was not assigned, the PT staffing schedule was reviewed on the relevant date to determine whether there was adequate staffing or not.

In order to assess the relationship between immediate PT and short-term outcome measures, a control group of 669 procedures that received immediate PT was identified by three-to-one matching for sex, age, BMI, ASA classification, insurance type, procedure performed, anesthesia type, operative time, and caseload. The control group was not matched for procedure start time due to its close correlation with delayed PT. The maximum allowable absolute difference between propensity scores was 0.01. Number of PT sessions prior to discharge, postoperative LOS, discharge disposition, and 30-day readmissions were compared between matched groups. Postoperative LOS was defined as the time from leaving the OR to discharge. For postoperative variables found to be different between groups, we used repeated-measures logistic regression to predict the outcome while adjusting for procedure start time and ability to ambulate on the same day as surgery.

Categorical data were analyzed with the chi-squared or Fisher's exact test (expected cell counts <5) and displayed using counts with percentages. Continuous variables were tested for normality using the Kolmogorov-Smirnov test. Normal data were analyzed with the two-sample* t*-test and expressed as means ± standard deviation, and nonnormal data were analyzed with the Mann-Whitney* U* test and expressed as medians with 25^th^ and 75^th^ percentiles. Logistic regression models were used to predict delayed PT based on both the operative start time and overall caseload by determining the start time and caseload that maximized the area under the receiver operating characteristic curve, which also maximized the sensitivity and specificity for determining delayed PT. All tests were two-sided and a p value < 0.05 was considered statistically significant. All statistical analyses were performed using SAS v9 (SAS Institute, Cary, NC).

## 3. Results

In total, 2051 primary TJA procedures were performed at our institution from July 2015 to December 2017. Of these, 226 (11.0%) did not receive immediate PT. These cases had a higher rate of general anesthesia (25.2% versus 17.8%, p=0.006), later median operative start time (13:26 [11:31-14:38] versus 9:36 [8:24-11:16], p<0.001), longer median operative time (1.8 [1.5-2.2] versus 1.6 [1.4-1.8] hours, p=0.002), and higher median overall caseload (6 [4-9] versus 5 [4-8], p=0.002) (Table 1). Data on operative time was not available for 66 patients and was excluded from analysis. The proportion of missing operative time data did not differ between groups (immediate PT: 3.2%, delayed PT: 3.5%; p=0.771).

A procedure start time after 11:30 (OR 12.09; 95% CI 8.71-16.77; p<0.001) and an overall daily caseload higher than six (OR 1.56; 95% CI 1.18-2.06; p=0.002) maximized the sensitivity and specificity of predicting delayed PT (procedure start time >11:30: sensitivity = 78.8%, specificity = 76.9%; overall caseload >6: sensitivity = 59.0%, specificity = 52.0%). Median time to first PT session was almost four times longer for the delayed PT group (18.4 [16.7-20.7] versus 4.8 [3.9-5.7] hours, p<0.001), and a lower percentage of these patients received their first PT session in the postanesthesia care unit (0.9% versus 18.4%, p<0.001). The most common reasons for delayed PT were missed PT assignment (n=62), late PT order activation (n=58), and failed attempted PT visit (n=41). The most common reasons for failed attempted visit were pain (n=20) and drowsiness (n=10). Postoperative complications (n=4) were a less common reason for delayed PT (Table 2).

Patients who received delayed PT were matched based on demographic and preoperative variables to a group of patients who received immediate PT. There were no differences in sex, age, BMI, ASA classification, insurance type, procedure performed, anesthesia type, operative time, or overall caseload between matched groups (Table 3). Patients who received delayed PT had a lower median number of PT sessions prior to discharge (3 [2-3] versus 3 [2-4], p<0.001) and an increased rate of unplanned 30-day readmission (7.6% versus 3.1%, p=0.004). No differences were observed in postoperative LOS or discharge disposition (Table 4).

Delayed PT, number of PT sessions before discharge, procedure start time, and ambulation on POD 0 were entered into a repeated-measures logistic regression model matched for baseline covariates to predict 30-day readmission. Delayed PT (OR 4.54; 95% CI 1.61-12.84; p=0.004) and number of PT sessions prior to discharge (OR 1.17; 95% CI 1.04-1.32; p=0.009) were associated with an increased 30-day readmission rate (Table 5).

## 4. Discussion

As perioperative care continues to become more expedited and health systems aim to implement programs to speed recovery and reduce costs, it is important to identify barriers to early rehabilitation and the potential impact on short-term outcomes. In this retrospective review of 2051 TJA procedures performed at a single institution, we found that the majority of patients (89%) received immediate postoperative PT. Patients who did not receive immediate PT were more likely to have a later procedure start time, general anesthesia, longer operative time, and a higher overall caseload on the day of surgery. We also found that patients who did not receive immediate PT had an increased rate of unplanned 30-day readmission.

This study is subject to the limitations of retrospective analysis. The clinical information collected is dependent on accurate and complete documentation. Also, there are multiple factors that can affect 30-day readmission following TJA [13–16]. We attempted to control for commonly reported factors, including age, sex, BMI, overall health status, insurance type, anesthesia type, operative time, length of stay, and discharge location; however, inclusion of other factors such as specific comorbidities and postoperative complications, which were not available for our study, would have strengthened our analysis. Along the same lines, some institutions schedule patients with higher medical comorbidity or surgical complexity near the end of the day, which could confound our results; however, the protocol at our institution is to schedule more comorbid and complex primary TJA patients at the beginning of the day, which makes this potential issue less likely. Finally, our study was performed using data from a single academic center, and results may not be generalizable to other regions, institutions, or practice settings.

Previous reports have noted later operative end time as a risk factor for not receiving PT on the same day as surgery [7]. Similarly, we found that patients receiving delayed PT were more likely to have a later operative start time. The odds of not receiving immediate PT were 12 times higher for patients with a start time after 11:30. Interestingly, the majority of delayed PT cases were the result of logistical factors such as late order activation, late anesthesia sign-off, missed PT assignment, or failure to see the patient. These reasons accounted for 80% of delayed PT cases, while patient-level factors such as refusal to participate in PT and postoperative complications accounted for only 20%. Longer operative time and higher total caseload were also associated with a decreased likelihood of receiving immediate PT. Altogether, these findings emphasize the importance of operative scheduling and PT staffing in the consistent provision of early postoperative PT. Indeed, one study [17] found that a simple change in PT staffing hours resulted in an increased proportion of patients receiving PT on the same day as TJA from 64% to 85%.

Another risk factor for not receiving immediate PT following TJA was general anesthesia. Multiple studies have demonstrated the benefits of regional over general anesthesia in TJA, including decreased LOS, reduced postoperative complications, and decreased 30-day mortality [18–23]. Our study suggests the additional benefit of allowing earlier rehabilitation following surgery, highlighting the interdependence of the various aspects of advanced perioperative care. This is consistent with results from a systematic review [24] that found that, compared to general anesthesia, regional anesthesia reduced postoperative pain and facilitated rehabilitation for patients undergoing TKA. We did not distinguish between various regional anesthetic techniques in our study, but it is important to note that growing evidence suggests that adductor canal block specifically, when compared to femoral canal block, is superior for postoperative rehabilitation and ambulation [25, 26].

With recent trends showing a decrease in LOS and an increase in 30-day readmissions for primary TJA patients [27, 28], there is concern that accelerated perioperative care regimens may increase the risk of short-term readmission. However, several studies indicate that this may not be the case [29–32], and others suggest that accelerated clinical pathways could even decrease early readmission following TJA [6, 33]. We found that immediate PT, an important component of accelerated perioperative pathways, was associated with a decreased 30-day readmission rate. Early mobilization following TJA has been associated with improvements in short-term functional outcomes, pain scores, and health-related quality of life [34]. It has also been associated with a reduced risk of deep vein thrombosis, infection, and gastrointestinal and pulmonary complications [35]. Interestingly, we found the relationship between immediate PT and 30-day readmission to be independent of ambulation during the first PT session, suggesting that there may be benefit to early individualized PT regardless of patients' postoperative abilities. Still, our results are based on retrospective analysis and require confirmation with high-quality, prospective randomized trials.

Several studies have demonstrated an association between earlier postoperative PT and shorter LOS [7–10]. Interestingly, we did not observe a decrease in LOS for patients receiving immediate PT following TJA. However, postoperative LOS was shorter overall in our study compared to previous studies, likely reflecting institutional differences as well as a continuing trend toward accelerated perioperative care and earlier discharge [6, 27, 28, 36]. Our median postoperative LOS was two days for both immediate and delayed PT groups. In comparison, Chen et al. [7] reported an average postoperative LOS of three days for patients who received PT on the same day as surgery and four days for those who did not. As LOS for TJA procedures continues to decrease with overall advances in perioperative care, the incremental effect of earlier PT may become less influential. Nonetheless, with the growth of outpatient joint arthroplasty [37], this study suggests that there may be value in receiving PT prior to discharge.

## 5. Conclusion

In conclusion, a majority of patients were able to participate in immediate postoperative PT following TJA. Barriers to receiving immediate PT included general anesthesia, later operative start time, longer operative time, and higher daily caseload. These factors present potential targets for improving the delivery of immediate postoperative PT. Early PT may help reduce 30-day readmissions, but additional research is necessary to further characterize this relationship.

## Figures and Tables

**Table 1:** Demographic and perioperative variables for immediate and delayed PT groups.

	Immediate PT	Delayed PT	p value
	(n=1826)	(n=226)	
Sex			
Female	53.3%	59.7%	0.065
Male	46.7%	40.3%	
Age	64.8±10.9	64.1±12.5	0.369
BMI	30.6±5.4	31.2±5.9	0.144
ASA			
I-II	44.2%	37.6%	0.061
III-IV	55.8%	62.4%	
Insurance Type			
Commercial	37.7%	33.6%	0.330
Medicare	58.5%	61.5%	
Medicaid	1.4%	2.7%	
Other	2.4%	2.2%	
Procedure			
TKA	63.2%	65.9%	0.418
THA	36.8%	34.1%	
Anesthesia Type			
General	17.8%	25.2%	0.006
Regional	82.2%	74.8%	
Median Procedure Start Time	9:36 [8:24-11:16]	13:26 [11:31-14:38]	<0.001
Median Operative Time (hours)	1.6 [1.4-1.8]	1.8 [1.5-2.2]	<0.001
Median Overall Caseload	5 [4-8]	6 [4-9]	0.002

**Table 2:** Reasons for delayed PT.

Reason	Number of Patients (%)
No PT Assigned	62 (27.4%)
Inadequate PT Staff	43 (19.0%)
Adequate PT Staff	19 (8.4%)
Late Order Activation	58 (25.7%)
Late Anesthesia Sign-Off	37 (16.4%)
Failure to See Patient	24 (10.6%)
Inadequate PT Staff	13 (5.8%)
Adequate PT Staff	11 (4.9%)
Failed Attempted PT Visit	41 (18.1%)
Pain	20 (8.8%)
Drowsiness	10 (4.4%)
Patient Unavailable	4 (1.8%)
Weakness	3 (1.3%)
Hypotension	2 (0.9%)
Nausea	1 (0.4%)
Language Barrier	1 (0.4%)
Post-Op Complication	4 (1.8%)

**Table 3:** Demographic and perioperative variables for matched immediate and delayed PT groups.

	Immediate PT	Delayed PT	p value
	(n=669)	(n=223)	
Sex			
Female	59.2%	59.6%	0.906
Male	40.8%	40.4%	
Age	64.3±11.5	64.1±12.5	0.787
BMI	31.2±5.5	31.1±5.9	0.773
ASA			
I-II	40.2%	37.7%	0.502
III-IV	59.8%	62.3%	
Insurance Type			
Commercial	34.1%	33.6%	0.895
Medicare	60.2%	61.9%	
Medicaid	2.5%	2.2%	
Other	3.1%	2.2%	
Procedure			
TKA	64.7%	65.5%	0.840
THA	35.3%	34.5%	
Anesthesia Type			
General	25.0%	24.7%	0.929
Regional	75.0%	75.3%	
Median Operative Time	1.7 [1.5-2.1]	1.8 [1.5-2.1]	0.420
Median Overall Caseload	6 [4-9]	6 [4-9]	0.448

**Table 4:** Postoperative variables for matched immediate and delayed PT groups.

	Immediate PT	Delayed PT	p value
	(n=669)	(n=223)	
Median Postoperative LOS (hours)	48.6 [28.6-54.8]	46.9 [30.5-68.8]	0.750
Median PT Sessions Before Discharge	3 [2-4]	3 [2-3]	<0.001
Mean PT Sessions Before Discharge	3.3±1.7	2.9±1.8	
Discharge Disposition			
Home/Self Care	62.5%	59.2%	0.819
Home Health Service	20.8%	24.2%	
SNF	15.2%	15.2%	
Rehab Facility	1.3%	1.3%	
Expired	0.1%	0.0%	
30-Day Readmission	3.1%	7.6%	0.004

**Table 5:** ORs for 30-day readmission.

	OR (95% CI)	p value
Delayed PT	4.54 (1.61-12.84)	0.004
PT Sessions	1.17 (1.04-1.32)	0.009
Procedure Start Time	0.93 (0.02-43.97)	0.969
Ambulation POD 0	2.02 (0.70-5.82)	0.192

## Data Availability

The data used to support the findings of this study are restricted in order to protect patient privacy. Data may be released upon application to the Duke Health Institutional Review Board.
